# Definitions of Ageing According to the Perspective of the Psychology of Ageing: A Scoping Review

**DOI:** 10.3390/geriatrics9050107

**Published:** 2024-08-23

**Authors:** Luca Gaviano, Roberto Pili, Andrea Domenico Petretto, Roberta Berti, Gian Pietro Carrogu, Martina Pinna, Donatella Rita Petretto

**Affiliations:** 1 Department of Pedagogy Psychology Philosophy, University of Cagliari, 09123 Cagliari, Italy; luca.gaviano@unica.it (L.G.); andreapetretto@yahoo.it (A.D.P.); roberta.berti80@libero.it (R.B.); gpcarrogu@gmail.com (G.P.C.); martina.pinna@hotmail.it (M.P.); 2 Worldwide Community of Longevity, 09124 Cagliari, Italy; dott.robertopili@gmail.com

**Keywords:** ageing, clinical psychology, conceptual models, Rowe and Kahn’s model, successful ageing

## Abstract

In the last decades, the scientific interest in ageing has increased due to the progressive ageing of the global population and due to the importance of guaranteeing the elder people and the next generations a good quality of life and biopsychological well-being. However, nowadays, there is not a common and accepted definition of ageing. This situation may refer to the complexity and relevance of the ageing topic and it means that the concept of ageing needs to be understood in a deeper way as a multidimensional and complex process that includes different elements distinctive. The main goal of this review is to explore the definitions and conceptual models of ageing according to a psychological point of view, through a biopsychosocial approach, that integrates biological, psychological, and social aspects with the main goal of a better understanding of the complexity of the process itself. Methods: We conducted a review of the literature through PubMed, Scopus, Web of Science and Google Scholar databases, and we followed the PRISMA-ScR guidelines, analysing papers written in English between 2002 and 2023. Results: The review showed different conceptual models of ageing, including Rowe and Kahn’s successful ageing model, the World Health Organization’s models, and others like the “Selection, Optimization and Compensation” Model. Also, the determinants and predictors of ageing have been analysed highlighting the individual variability and the multidimensional nature of ageing. The geographic diversity of the included studies allowed for the analysis of cultural, socioeconomic, and environmental differences in the conceptualisation of ageing. Conclusions: The results emphasise the importance of targeted interventions and programs aimed at promoting well-being during ageing, considering the complexity and multidimensionality of the ageing process.

## 1. Introduction

In the last decades, the scientific interest in the topic of ageing has increased significantly, also thanks to the progressive ageing of the population. Some approaches attempt to study the ageing process of the population by adopting a biopsychosocial model, which considers together the biological, psychological, and social aspects and the interrelationship between them [1,2,3,4,5,6]; moreover, an increasing number or researchers focused on psychological point of view, and they have developed many different conceptual models of ageing. According to those approaches and researchers, ageing is a complex phenomenon, with positive and negative aspects, and it is also a phase in the life span, the more advanced phase or (phases). Starting from this theoretical position, and from a biopsychosocial point of view, it is very important to gain more knowledge about what it means to “age well” and to gain a shared definition. However, nowadays there is still an open debate, and a shared scientific definition of ageing from a psychological point of view is not available [3,4,5,7,8,9]. Because of this debate, in the international literature of ageing, we found different terms and adjectives associated with the word “ageing” as: “successful”, “well”, “usual”, “active”, “positive”, “healthy”, “productive”, “good”. Each of these terms tends to focus on “what ageing means” and more in general “on what living well and living long means”. Some authors have used some specific keywords “quality of life” and “wellbeing”, and there are some other factors that could be considered when we investigate the ageing process from a psychological point of view; it means that we would also consider the multiple dimensions/factors of human life experience during ageing [1,7,8,9]. This exploration encompasses several key aspects. Firstly, individual variability plays a significant role: ageing involves multiple dimensions, including biological, psychological, and social factors [2,6,10,11]. However, how individuals navigate this phase of life is unique and influenced by their life experiences [12]. Secondly, ageing is a multidimensional process, representing a complex phenomenon within the circle of life. It encompasses not only biological aspects but also psychological, social, and emotional dimensions [13]. Thirdly, stereotypes and expectations surrounding ageing can significantly influence its definition and psychological perception [14]. Lastly, cultural influences shape the understanding and interpretation of ageing across different societies and communities [2,4,6,10,15,16]. While the debate is still open and there is considerable diversity in semantic perspectives, the increasingly ageing population necessitates mandatory implementation of interventions and programs, particularly in the realm of promoting well-being in ageing. Consequently, researchers acknowledge the imperative to explore and analyse variables and determinants that facilitate the promotion of optimal ageing processes.

Bearing in mind those aspects and aiming to offer a contribution, in this paper, we aim to review the literature about ageing from a psychological point of view, focusing on the following four research questions:

a. What definition (s), and model (s) were previously used to describe ageing from a psychological point of view according to the bio-psycho-social approach?

b. In which way did the previous paper describe the role of the variables as predictors or determinants?

c. Did previous studies focus more on the theoretical model(s) to study the psychological construct of ageing, or did they start from lay perceptions of ageing people?

d. Regarding the age of samples, did previous studies focus more on the perception of people in the more advanced phases of life or did they consider the perception of people in all the phases of life to acquire information for both cases?

## 2. Methods

### 2.1. Protocol

The protocol was developed using the scoping review methodological framework proposed in PRISMA-ScR Guidelines [17]. We reported data according to these guidelines. We conducted a literature review about the definition of ageing from a psychological point of view, using different electronic databases such as PubMed, Scopus, Google Scholar, and Web of Science. All studies eligible, including those that utilised qualitative and quantitative methods, methodology or guidelines report, except reviews and metareviews. We also excluded papers written in other languages than English. No time limits were considered, but according to the chosen topic, we found articles from 2002 until 2023.

### 2.2. Information Sources and Search Strategy

Literature searches were conducted in the following online databases: Scopus, PubMed, Google Scholar, and Web of Science. These databases were chosen to cover health sciences. We used the following search keywords: “Ageing/Aging” “Definition/Definitions” “Psychology” combined with the “AND/OR” Boolean operations. The literature was selected, and results were analysed. A total of 2914 records were found, and the keywords were searched in the publication title or abstract. 

**Figure 1:**
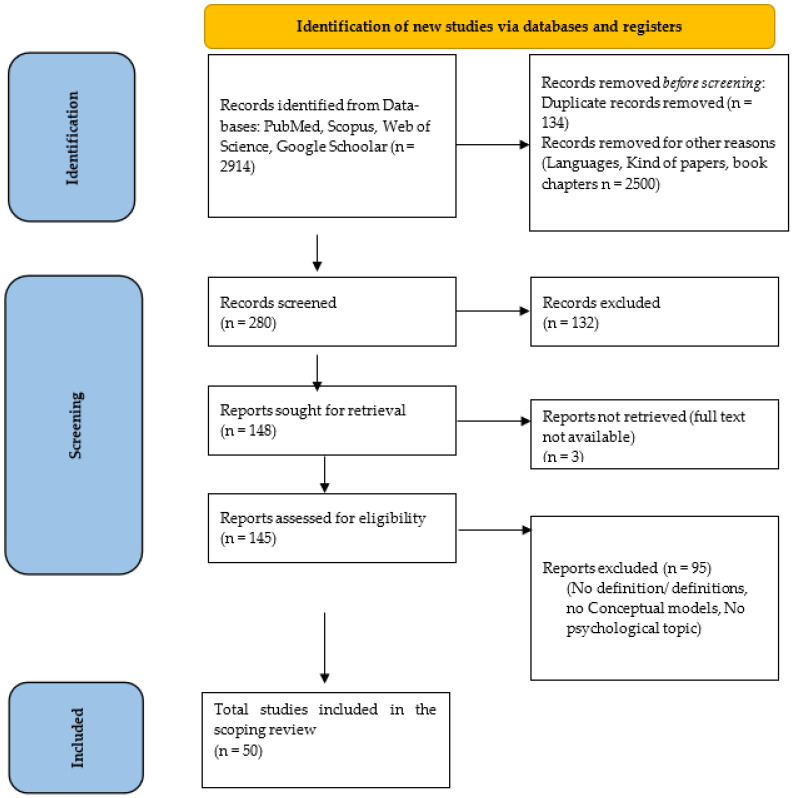
Prisma-SC PRISMA 2020 flow diagram for updated systematic reviews which included searches of databases, registers, and other sources. From: https://www.prisma-statement.org/scoping, accessed on 12 August 2024.

As Figure 1 shows, we had those results.

LG and DRP independently reviewed the chosen references, deciding to exclude further papers. A total of 148 papers were found. Papers were analysed with respect to their content, and papers with content that was not fully within the scope of this review were eliminated. Starting from the references in full text of the articles derived from the literature review, some other papers were excluded. Then the duplicate references were removed. After the reading of the full text, a total of 50 papers were then considered for the final analysis.

### 2.3. Methodological Quality Appraisal

According to Tricco and colleagues [17] and considering the peculiarity of scoping review, we did not appraise methodological quality or risk bias of the included papers. After examination of includes articles and according to the quality of the studies and the research questions, we did both quantitative and qualitative analyses of the papers. 

## 3. Results

This scoping review aims to provide a comprehensive overview of conceptual models and definitions of ageing according to the perspective of psychology, as well as determinants and predictors of ageing analysed in the works under consideration, following an examination of various parameters including the language used, temporal nature, and geographical distribution of the selected articles. 

During the article selection process, a rigorous exclusion was applied to those not available in English. This criterion was adopted to ensure linguistic coherence and facilitate understanding, and data synthesis. Moreover, the adoption of English enables greater accessibility and sharing of sources and materials used in the scoping review.

To strive for a more complete and updated view of the field, the research focuses on articles published between 2002 and 2023 to understand the evolution of perspectives on successful ageing through a biopsychosocial approach. This timeframe was chosen to provide a comprehensive overview of developments in the field of ageing psychology over the past twenty years.

One of the significant perspectives of this scoping review is the geographical diversity of the included studies, focusing on the concept of successful ageing. This diversity allows examining cultural, socio-economic, and environmental differences that may influence different conceptualisations. Articles were carefully selected to represent a wide range of geographical contexts, ensuring a comprehensive and inclusive view of research conducted worldwide. Table 1 shows the geographical distribution of the sorted papers. 

Among the geographical areas covered are:

Asia: Bangladesh, China, India, Korea, Malaysia, and Taiwan [18,19,20,21,22,23,24,25,26,27]. 

Europe: England, France, Finland, Germany, Greece, Italy, Norway, Poland, Portugal, Spain, Sweden, and Wales [5,28,29,30,31,32,33,34,35,36,37,38,39,40,41,42,43,44]. 

America: Alaska, Latin America, Canada, Chile, and the United States [45,46,47,48,49,50,51,52,53,54,55,56,57,58,59,60,61]. 

Australia: [62,63,64,65,66]. 

The table (Table 2) shows the results: 

In the following, we will discuss each specific research question. 

a. What definition, definitions, model/models were previously used to describe ageing from a psychological point of view according to the bio-psycho-social approach?

To answer the first research question, we have analysed the selected papers and we observed that the more widespread model/definition is the one related to “Successful Ageing model”, proposed by Rowe and Kahn [67,72]. This model is based on the first definition of “successful ageing” proposed by Havinghurst in 1961 [68]; some authors proposed various minor and major changes, according to specific critical loci described by other specific research. From a general point of view, the Rowe and Kahn’s model (Successful Ageing) has been described in almost all the selected papers [5,18,19,20,21,22,23,24,25,26,27,28,29,30,31,32,33,34,35,36,37,38,39,41,42,43,44,46,47,48,49,50,51,52,53,54,55,56,57,59,60,61,62,63,64,65,66]. 

Some other models have been proposed, like the World Health Organization models, Active Ageing and Healthy Ageing [19,26,29,37,38,39,40,43,52,71,82], The Communicative Ecology Model of Successful Ageing [45,70], Ageing Well [5,18,39,58,59,66,68], The Selection, Optimization, and Compensation (SOC) Model [12,19,22,27,33,37,41,45,51,54,57,58,78], Lay Perspective on Successful Ageing [12,22,23,24,26,29,30,31,34,37,38,42,51,58,59,74,92], Robust Ageing [34,75], The Disengagement Theory [5,43,63,76], Psychological well-being [63,74,92], Positive Ageing [38,39,40,74,85,92], Usual Ageing [23,34,52,57,63,66], Productive ageing [40,43,52,84,93] and Two Factors Model of Successful Ageing [61,90]. 

In the following, we will briefly describe each model, and we will describe the proposed definition(s) given in each model. 

### 3.1. Successful Ageing 

The concept of “Successful Ageing” (SA) has been investigated by many researchers during the last decades. In the first step, the idea of “successful ageing” was related to one person’s level of activity in his/her life during the more advanced phase(s) of his/her life. 

#### 3.1.1. Successful Ageing Model by Havinghurst [68] 

The term “Successful Ageing” was introduced, for the first time, in 1961 by Robert J Havinghurst, with the practical intention to identify a model that could “add years of life” by increasing satisfaction and well-being during the later stages of life circle [68]. Havinghurst, himself, understood that this could be a very complicated task because the theorisation of the model involved different multidimensional variables such as biological, psychological, cognitive, physical, affective, and social ones. 

Many of the first definitions of “Successful Ageing” have been conceptualised by the biomedical model, which defines them on the dichotomic basis of disease or health. Considering this, “successful ageing” has been defined as the presence of good quality of health, independence, high cognitive functional levels and the absence or reduction of risk factors that could evolve into disabilities or diseases. 

Soon after, the authors realised that there were deep interconnections among bio-psycho-social factors in determining the health condition of the person (a new model defined as biopsychosocial, was proposed in which the concept of health is described as a spectrum that derives from multiple dimensions [94,95]). 

#### 3.1.2. Successful Ageing Model by Rowe and Kahn [67,72]

The biopsychosocial model has been added and included in various definitions and models, but the most well-known model is the “Successful Ageing” by Rowe and Kahn [67,72].

The first model of “Successful Ageing” was proposed for the first time in 1987. The authors believed that the role of the losses in the ageing process is often overestimated and that the age-related decline could be explained in terms of lifestyles, habits, diet, and extrinsic psychosocial factors. In addition, they believed that the previous perspective did not consider the impact of the extrinsic variables and the interrelationship between the psychological and social variables during the ageing process. They also introduced the term “Usual Ageing” to refer to the process of ageing that resulted in challenges and typical shifts that occurred with ageing, involving the state and functionality of health without necessarily achieving optimal levels of health and well-being. This approach underlines that despite the challenges and losses, it is possible to reach a good level of well-being. They opposed the concept of “Successful ageing” in which they emphasise the importance of maintaining a good quality of physical, mental, and social health during the ageing process. Their model received some criticism; one of the major criticisms is mainly related to the terminology used, particularly the term “success”. Some authors argue that the use of this term does not consider the unique challenges that many people have had to face during their ageing process [67].

In 1997, Rowe and Kahn revisited their work from 1987, and they made some changes. These authors defined and conceptualised “Successful Ageing” as the result of three components or constructs: the absence of/low probability of disease, high physical and cognitive functioning, and engagement in activities of daily life. 

The first component was defined as the absence or presence of low-risk factors for certain diseases such as various cardiovascular problems, chronic bronchitis, cancer, diabetes, depression, etc. The second component was defined by the two authors as being able to independently perform certain daily life tasks such as: going to the bathroom, getting out of bed, and performing simple household chores (e.g., washing dishes, doing the laundry) or other specific potential tasks; these indicate what a person can or can’t do. Finally, the third component involves being engaged in relationships with others and being productive in society. Relationships involve emotional support and information exchange, but also the need for assistance. Productive activities are considered as “generating value” which can or cannot be remunerated (as in the case of volunteering) [72]. Furthermore, the authors highlighted that successful ageing is not determined only by the individual elements described but it is the continuous interaction among these just described three components that determines successful ageing: the two authors wanted to emphasise the importance of approaching ageing with a combination of health maintenance, active engagement, and a positive attitude toward life. This approach stands out for its focus on the quality of life in ageing rather than just the challenges and losses. 

#### 3.1.3. The Communicative Ecology Model of Successful Ageing (CEMSA)

This model proposed by Fower, Gaisorek and Giles [70] is based on a theoretical approach which sees communication as a factor that could influence the successful ageing process. This model aims to better understand how social interactions and communication may contribute to well-being and to the quality of life. Older people may need to feel themselves involved as part of social environments, which promote interaction. The way and the results of those relations determine the perception of well-being, the quality of relationships and the ageing process in elder people. According to this model, there can be some different communicative social environments, such as relationships with peers’ family members, friends, and local communities. Moreover, relationships with the organization, social services and social health can have a different impact on the perception of an individual’s ageing process [45,70]. Furthermore, all the opportunities for exchange and communication that have developed with the advance of technological progress may allow the elderly to develop new modes of communication that may then turn into social and learning opportunities [45,70].

#### 3.1.4. A Two-Factor Model of Successful Ageing 

Pruchno, Wilson-Gerderson and Cartwright [90] proposed a model of successful ageing based on two main factors that, according to the authors, contribute to achieving successful ageing [61,90]. This model seeks to understand how people can reach a good quality of life and a state of well-being during the ageing process through the two mentioned factors. The first factor, namely the objective ones, refers to Health and Functional Capacity, that is, physical and functional abilities. It is based on the idea that health and the ability to perform activities of daily living independently are fundamental elements for successful ageing. This factor includes other processes within it, such as maintaining a healthy lifestyle, preventing the onset of diseases, and managing existing health conditions. The second factor, the subjective one, refers instead to Psychosocial Functioning. This dimension focuses particularly on mental, social, and emotional well-being. It includes factors such as the creation of satisfying social relationships, and adaptation to the psychological challenges noted in this life cycle. Social support, engagement in social activities, and the creation of interpersonal relationships are important aspects of the psychosocial well-being of the individual. This model emphasised that both physical health and psychosocial well-being are interconnected and complementary in determining successful ageing. Furthermore, it is recognised that successful ageing is not only determined by physical health but also incorporates psychological, emotional, and social factors [61,90]. 

### 3.2. The Health, Positive and Successful Ageing 

The model proposed by Fernandez-Ballesteros [92] represents the European response to the Rowe and Kahn model [96]. According to the author, successful ageing consists of good physical functioning, high cognitive levels, positive emotions, and good levels of social participation. The outcome of successful ageing is derived from life experiences, personal beliefs, and surrounding environment in which the individual is placed. Her perspective helps to destroy the myth that ageing is only a phase of decline and instead highlights how the ageing process can offer opportunities for growth, adaptation, and personal realisation [92,96]. 

Next to the concept of successful ageing, the author proposes the conceptual model of positive ageing, postulated in three key points. The first emphasised that ageing should be understood as a complex factor capable of manifesting itself differently both qualitatively and quantitatively. The second aspect concerns the individual capacities that individuals have maintained throughout their lives and that also promote renovation and optimisation of their resources during the ageing process. The third point addresses the issue of losses; the author argues that, of course, individuals experience losses in ageing, but these can be compensated through targeted learning and change processes to balance the loss itself [92]. Positive ageing aims to be a new way of perceiving the ageing process, no longer as negative but adopting a large and more realistic view that focuses on compensation and change to maintain a high level of personal satisfaction [92].

### 3.3. World Health Organization’s Models

#### 3.3.1. Active Ageing

In 2002, the World Health Organization (WHO), defined the concept of “Active Ageing”, as “a process of optimizing opportunities for health, participation, and security to improve the quality of life for ageing individuals” [71]. This model tends to emphasise the strong positive correlation between ageing actively and the benefits that occur on both physical and psychological levels, leading to an increase in the quality and level of life satisfaction. However, the term “active” does not refer to a vision of individuals over 65 as active physically and occupationally, but to a perspective that values their continuous participation in the economic, social, cultural, and religious world and the maintenance of their role in their own context of belonging. The model of active ageing indicates, therefore, the way in which the individual carries out activities for which they decide to engage based on their aspirations [71].

#### 3.3.2. Healthy Ageing 

This second concept of ageing proposed by the WHO, following the criticism received in the previous model, focuses on the development of individuals’ functional capacities in environmental contexts which promote interaction between the individual’s capabilities and the environment itself [97]. This model, the so-called “Healthy ageing” model includes the need to consider the heterogeneity of experiences that may be relevant for all ageing individuals, regardless of their health status and the idea that older people can adapt and shape their lifestyles based on the challenges they may address. The WHO has, in fact, defined healthy ageing as “the process of developing and maintaining functional abilities that enable individuals to achieve the desired level of well-being in old age” [97]. It is a structure model, based on the continuous interaction between three fundamental elements: functional abilities, intrinsic capacities, and the environment [97]. Functional abilities correspond to the health condition of the individual that allows them to do and be what they desire. They refer to all the skills that an individual employs to perform tasks, face challenges and achieve their goals. Intrinsic capacities refer to all the physical and mental capabilities that an individual has throughout their life; these tend to decrease over the course of life, and this reduction can result from various factors and is more noticeable in old age. The environment consists of various contexts that are crucial in an individual’s life, identifying and characterising them based on their level of belonging; these contexts include the family, social–healthcare assistance, school, social policies, and work. The purpose of this model revolves around three fundamental elements: postponing the onset of chronic disease, preventing potential relapses and exacerbations in individuals with an already established disorder and preserving and increasing the autonomy of the elderly [97].

### 3.4. Productive Ageing

In 1969, Butler coined the term “ageism” in his work titled: “Ageism: another form of Bigotry” to analyse the phenomenon of age-based discrimination. His work highlighted how the elderly are treated negatively and stereotypically in society. For this reason, he explored the concept of “Productive Ageing” linking it to the concept of “Active Ageing” to dispel these negative stereotypes about the elderly and promote active and participatory ageing [93]. 

He emphasised the importance of allowing the elderly to continue making an active contribution to society through social, economic, and cultural involvement. This involvement is determined by various aspects, including social participation, meaning taking part in the social life of the community in which the elderly person is integrated; productive activities, involving engagement in activities such as paid work or volunteering; learning and development, understood as individuals’ ability to continue learning, acquire new knowledge and skills, and update previous skills; and maintaining health, with Butler stressing the importance of taking care of one’s health through a healthy lifestyle. 

Some years later, this model was revisited in a positive manner by Gleason in 1985, with the aim to promote active and participatory ageing that contrasts with the negative view of ageism, and to provide a more positive perspective on ageing as a life stage full of opportunities and respect [93,98]. 

### 3.5. Positive Ageing: The Life Span Diamond

The “Positive Ageing” model proposed by Gergen and Gergen in 2006 [99], promotes a proactive and positive attitude toward ageing, emphasising the possibilities for growth, satisfaction, and well-being. They describe it as the “diamond of the life arc” because it not only speaks about well-being throughout the course of life but also because its starting points encourage thinking in terms of continuous life enrichment. The four points that constitute this diamond are: (1) Relational Resources: social involvement is considered essential for positive ageing; maintaining connections with friends, family and the community contributes to a sense of belonging and support; (2) Physical Well-Being: taking care of one’s health is emphasised with a healthy lifestyle determined by psychical exercise, a balanced diet, and stress management practices being fundamental pillars of this model; (3) Engaging Activities: positive ageing highlights the importance of continuing to participate in activities that bring satisfaction and a sense of accomplishment, such as volunteering, learning new activities, and engaging in hobbies; (4) Positive Mental States: this approach explores psychological and emotional well-being in the elderly. It involves cultivating positive emotions, resilience, and the ability to successfully face challenges with optimism. This allows for two fundamental outcomes: personal fulfilment and the ability to adapt to changes [99]. 

An essential aspect of this model is the relationship among the four described points. Indeed, there can be bidirectional exchanges among these points; for example, positive mental states can lead to physical well-being, and physical well-being can simultaneously contribute to positive mental states. Positive ageing is thus a paradigm that contrasts with the idea of ageing as a period of decline and promotes an optimistic perspective that recognises the potential for personal growth, achievement, and satisfaction even in old age [99]. 

### 3.6. The “Selection, Optimization, and Compensation” SOC Model

The “Selection, Optimization, and Compensation” (SOC) Model is a theoretical approach promoted by Baltes and Baltes and their research team in the field of developmental psychology. This model explores how individuals cope with challenges related to ageing and functional losses. It suggests that older adults face ageing-related changes through three main processes: selection, optimization, and compensation [12].

Selection: older individuals seek to select activities, goals, and challenges that are most meaningful and adaptable to their abilities and resources. In practice, they choose to focus on what is most important and achievable, abandoning, or deducing activities that might require excessive resources and skills. 

Optimization: this process involves a continuous effort to optimise the remaining personal resources. It means developing and maintaining skills, knowledge, and resources to effectively confront the challenges of ageing. Older individuals seek ways to adapt to new circumstances and learn new strategies to maintain optimal functioning.

Compensation: when personal resources decline or challenges become more difficult to handle, older individuals seek to compensate for these losses through the use of alternative strategies. These strategies may involve the use of tools, technologies, or engaging external resources such as the social support network. 

Therefore, the SOC model provides a perspective on how older individuals can adapt and cope with ageing-related changes. This model highlights the importance of making conscious choices, optimising resources, and compensating for functional losses to improve the quality of life in the later stages of adulthood [12].

### 3.7. The Proactive Coping Model 

The Proactive Coping Model focuses on managing stress and challenges in life. It is based on the idea that people can better cope with difficult situations when they adopt a proactive rather than a reactive approach. Instead of responding passively to stressful events, the model encourages individuals to take initiative and adopt active strategies to deal with problems. This approach involves several phases [100,101]: (1) Anticipation: recognising that stressful events are inevitable in life and anticipating the challenges that may arise; (2) Preparation: getting ready in advance by developing personal, social, and cognitive resources that can be used to cope with future stresses; (3) Proactive Coping: actively facing stressful events with a sense of control. This may involve using problem-solving strategies, seeking social support, and modifying one’s emotional reactions; (4) Reconstruction: after coping with stress, reflecting on the experience and learning from it to improve proactive coping strategies for facing future events [100,101]. This model aims to promote mental well-being and resilience by encouraging individuals to become active in managing life challenges rather than feeling helpless or overwhelmed [100,101].

b. In which way did the previous papers describe the role of the variables as predictors or determinants?

To reply to second research question, in the psychological models of ageing, we will look at the concepts of determinants and predictors which play distinct roles in understanding the ageing process, its trajectories, and its psychological implications. As people get older, psychologists study the complex aspects that shape this process. 

### 3.8. Determinants and Predictors

Determinants encompass the foundational elements that significantly impact the ageing trajectory. From a psychological point, these determinants may include genetic predispositions, cognitive frameworks, life experiences, and psychosocial influences. For instance, mental resilience, fundamental cognitive abilities, and effective stress management are recognised as psychological determinants that intricately influence the perception and experience of ageing [102].

On the other hand, predictors serve as invaluable tools in anticipating or estimating specific outcomes associated with ageing. In the psychological realm, these predictors may span measures of mental health, cognitive functioning, psychosocial adaptation, and social support. Robust social networks, regular cognitive engagement, and adaptive coping strategies are considered positive predictors associated with healthier and more resilient ageing trajectories.

The interplay between determinants and predictors unfolds as a complex mix involving biological, psychological, and social factors. A biological determinant, such as genetic predisposition, may influence cognitive functioning (predictor), while life experiences and social support (determinants) modulate the impact of these predispositions.

A psychological exploration of ageing through the lenses of determinants and predictors offers a comprehensive framework to understand the multifaceted nature of the ageing process. While determinants lay the groundwork for the ageing experience, predictors provide insights into anticipating individual variations in this intricate journey. Integrating these perspectives enriches our understanding of the psychological dynamics inherent in the process of ageing in the psychological realm [102].

### 3.9. Social–Demographic Characteristics

Socio-demographic variables—such as age, gender, education, income, ethnicity, marital status and place of living—act as important parts of a puzzle, that helped us to frame in a general way certain factors that may play an important role in understanding some characteristics of the participants in an empirical study [5,21,22,23,25,26,32,38,40,41,42,50,55,57,59,61,102]. Many of the articles analysed for this scoping review have in common in their respective studies, the analysis of socio-demographic variables in the study of ageing. These variables include a number of factors related to the social and demographic context of individuals, providing researchers with valuable information on the context of their study. 

Below is a summary of the factors most frequently cited in the various studies:

Age: One fundamental socio-demographic variable is age. Age can influence cognitive development, behavioural patterns, and life experiences, all of which are vital considerations in psychosocial studies. Researchers often categorise participants into different age groups to explore how psychological phenomena vary across the lifespan.

Gender and Sex: Gender and sex are critical variables that contribute to the understanding of psychosocial processes. Exploring the differences and similarities between males and females can uncover valuable information about the impact of gender identity on mental health, social interactions, and overall well-being.

Education Level: The level of education attained by participants is another key socio-demographic variable. Education can influence cognitive abilities, problem-solving skills, and access to information, all of which may impact psychological processes. Researchers often examine how education levels correlate with various psychological outcomes.

Income and Socioeconomic Status: Income and socioeconomic status provide insights into the economic context of participants. These variables can influence access to resources, opportunities, and stressors, thereby affecting mental health and behaviour. Understanding the role of socioeconomic factors is crucial in developing interventions and policies to address psychosocial well-being.

Ethnicity and Cultural Background: Exploring participants’ ethnicity and cultural background is essential for understanding the cultural context that shapes psychosocial processes. Cultural factors influence values, beliefs, and social norms, all of which contribute to variations in psychological experiences across diverse populations.

Marital Status and Family Structure: Marital status and family structure are socio-demographic variables that impact social support systems and interpersonal relationships. Researchers examine how these factors influence mental health, coping mechanisms, and overall psychological resilience.

Geographic Location: The geographic location of participants is considered to understand regional variations in psychological phenomena. Cultural differences, access to mental health resources, and environmental influences can all vary based on geographic location [5,21,22,23,25,26,32,38,40,41,42,50,55,57,59,61,102].

In the following section, we will analyse in a deeper way predictors and determinants. 

### 3.10. Education or Schooling

Among predictors, education or schooling is referred to as one of the most important [23,25,26,28,31,32,34,38,39,40,41,42,43,46,50,52,53,55,58,59,61]. As a result, the greater the number of years of schooling of a person, the greater the chances that this maintained high cognitive functions [103,104]. It therefore emerges that education is to be considered as an important protective factor against the deterioration of cognitive functions. Two possible explanations have been given to this shared statement: The former argued that the more people achieve high levels of schooling, the more likely they were to develop advantages in terms of improving neural circuits and cognitive functions, which was in line with what Rowe and Kahn argued [72]. The second was that education should be understood as a life-long intellectual activity that enables all people, even at the most advanced stages of the life cycle, to acquire new skills, knowledge, and abilities, and that could serve to avoid the process of deterioration of cognitive functions, as explained in the SOC model [12].

### 3.11. Physical Activity

Physical functioning is one of the most dominant themes in studies of successful ageing [18,19,20,22,23,24,26,27,29,30,32,33,34,38,44,46,47,48,50,51,52,53,55,57,63,79].

Good health is an essential part of a successful old age and in general, is a highly important issue. In addition, being healthy also enables one to be autonomous, which is another important component of successful and good ageing. Avoiding a sedentary lifestyle and maintaining robust physical health, strength, and energy emerged as pivotal aspects of successful ageing. Most older individuals believed that maintaining good physical health depended on being proactive in preventing serious health issues.

Physical activities contribute to reducing the risk of several diseases associated with ageing including cardiovascular diseases, metabolic disease, and osteoarthritis. According to the American College of Sports Medicine guidelines moderate weekly physical activities (30 min, 5 days/week) are recommended to obtain healthy benefits [105]. Many studies demonstrated a relationship between higher levels of physical activity and lower levels of cognitive decline. Moreover, exercise in mid-life and late life was associated with a reduced risk of mild cognitive impairment [106]. 

### 3.12. Cognitive Functioning and Cognitive Stimulation/Remediation

Maintaining mental health and cognitive abilities emerges as fundamental for optimal ageing [5,20,21,22,23,26,28,30,31,35,38,39,50,52,58,60,61,62,66]. Throughout numerous interviews, participants underscored the significance of preserving cognitive function and warding off conditions like dementia. Many contrasted themselves with peers experiencing memory issues, highlighting the value of staying mentally active, continuously learning, and retaining strong memory capabilities. It has been demonstrated that cognitive stimulation may reduce the onset of cognitive disease, and it may delay cognitive decline. For those reasons, many elders are being encouraged to practice cognitively stimulating activities in daily life. Cognitive interventions can be very helpful in improving various aspects of objective cognitive functioning including memory performance, executive functioning, processing speed, attention, fluid intelligence, and subjective cognitive performance. 

### 3.13. Diet and Nutrition

Dietary and nutritional interventions are among the most studied strategies for extension of the lifespan and prevention of morbidity. There is some evidence that obesity is associated with a heightened risk for dementia when individuals are followed longitudinally. The Mediterranean diet has shown associations with reduced rates of depression and lower risk for cognitive decline. Adherence to the Mediterranean diet includes a high consumption of fruit and vegetables, a high ratio of polyunsaturated to saturated fats and a low glycaemic load. Frontiers in the understanding of diet and ageing include research on the relationship between genetic risk and nutrition, as well as the intersection of the gut microbiome with mood and anxiety symptoms [5,25,26,38,42,47,53,57,60,107].

### 3.14. Social/Community Engagement 

The association between social engagement, health and well-being has been well-documented throughout the lifespan. In many ways, increased age can be considered a risk factor for social withdrawal, as a result of physical decline and retirement. Moreover, social engagement was as strong a protective factor for health as many other established risk factors. Social engagement can be defined as making social and emotional connections with other people such as family, friends, and the community. Community engagement provides seniors with a sense of purpose and a role in the community. The quality of their life corresponds directly to the quality of their social network. The community’s interaction demonstrates that the elders desire to be involved and the community should provide activities and opportunities that engage the elders. Supporting the role of elders in their community helps them to increase their sense of generativity. For the elders became very important to passing their knowledge, their wisdom and experience to the young [5,19,20,21,22,23,24,25,28,30,31,32,34,35,36,38,41,43,44,47,48,53,55,56,60].

### 3.15. Emotional Well-Being/Health

Emotional well-being for elders refers to the overall state of their mental and emotional health, including feelings of contentment, happiness, and fulfilment as they age [5,21,24,25,26,48,49,53,61]. It involves having a sense of purpose, meaningful connection with others, and the ability to cope with life’s challenges and changes effectively. Emotional well-being in elders may also involve a sense of resilience, adaptability, and acceptance of the ageing process, as well as the ability to maintain positive relationships and engage in activities that bring joy and satisfaction. It can vary among individuals but generally reflects a sense of inner peace, balance, and psychological stability in later stages of life. The elders’ positive emotional well-being is maintained because they believe in improvement and have goals to facilitate it. They engage in behaviours such as serving as traditional leaders and teachers serving as a positive influence on the youth. 

### 3.16. Resilience and Adaptation

Within the realm of “Resilience and Adaptation” individuals demonstrated emotional mastery and the ability to avoid persistent negative moods [12,22,32,39,51,54,56,61]. Psychological resilience and the capacity to adjust to novel circumstances were integral. Elders perceived themselves as ageing “successfully” when they derived pleasure, satisfaction, and fulfilment from daily experiences, navigating changes in health. Adaptation entailed reevaluating or redefining objectives and was deemed an unavoidable and pivotal aspect of ageing gracefully in the presence of age-related diseases, disorders, and disability.

### 3.17. Spirituality/Religiousness

The research highlighted the importance of spirituality or religion in the concept of successful ageing as a protection factor in the ageing process and in maintaining a healthy lifestyle [18,23,25,27,36,38,46,49,53,57,58,59,60,61,64,108]. According to Crowther and colleagues [108], spirituality among older adults has not been fully integrated into intervention models promoting successful ageing. However, they found spirituality is associated with improved well-being, reduced depression, morbidity, and increased longevity among older adults. Crowther et al. [108] expanded Rowe and Kahn’s model of successful ageing by including spirituality, recognising its significance in many older adults’ lives, indeed many elders explicitly recognise spirituality’s positive effects on their health, they mentioned how it alleviated worries. Spirituality and religiousness could be significant for the elder’s health and well-being. While attending church was one way, they socialised and remained active, spirituality for them extended beyond church attendance. Many elders described being spiritual throughout the day praying for their family and community. Another important consideration is that church attendance varied among the communities. 

### 3.18. Autonomy/Independence

Independence was recognised as a crucial aspect of successful ageing [20,29,33,35,36,40,55]. This encompassed maintaining a sense of personal control, self-assurance, and the freedom to make decisions as individuals grew older. It is noteworthy that the autonomy described by participants differed from mere “independence”. Instead, it emphasised the ability to make choices regarding social interactions (such as guiding caregivers), engaging in meaningful activities, and selecting suitable adaptive tools. Often, there was a strong focus on the significance of having choices and feeling empowered to decide how to participate in meaningful activities within one’s capabilities. Autonomy also involves the capacity to lead and actively engage in one’s caregiving and to have a say in matters like housing and other important decisions. Discussions on autonomy frequently intertwined with those about resilience and adaptability, highlighting the value of personal choice in adjusting to changing situations [20,29,33,35,36,40,55]. 

### 3.19. Financial Security/Financial Independence

As individuals progress through the stages of life, the role of income becomes increasingly significant, particularly in the context of successful ageing [5,18,20,24,25,26,27,29,33,37,40,42,53,54,60]. Successful ageing encompasses various dimensions including physical health, mental well-being, social engagement, and financial stability. While income alone does not guarantee successful ageing, it serves as a critical factor that can significantly influence one’s ability to navigate the challenges and opportunities associated with growing older. Income provides a sense of financial security that forms the foundation for successful ageing. Adequate income enables access to essential resources such as quality healthcare, nutritious food, comfortable housing, and opportunities for leisure activities. These resources play pivotal roles in maintaining physical health, cognitive function, and overall quality of life as individuals age. Moreover, financial stability offers a sense of reassurance and peace of mind, alleviating stress and anxiety related to economic uncertainty. Income also facilitates active participation in societal activities and fosters social connections, both of which are integral components of successful ageing. Older adults with higher incomes often have greater opportunities for social engagement, including participation in community events, leisure pursuits, and meaningful social interactions. These activities contribute to a sense of belonging, purpose, and fulfilment, which are fundamental aspects of psychological well-being in later life [5,18,20,24,25,26,27,29,33,37,40,42,53,54,60]. Furthermore, income affords older adults the flexibility to adapt to changing circumstances and pursue opportunities for personal growth and enrichment. Whether it involves exploring new hobbies, travelling to new destinations, or pursuing lifelong passions, financial resources provide the means to embrace new experiences and maintain a sense of vitality and enthusiasm for life. However, it is essential to recognise that income inequality and socioeconomic disparities can pose significant barriers to successful ageing for many individuals. Those with limited financial means may face challenges accessing essential services, maintaining adequate living standards, and participating fully in social and cultural activities. Addressing these disparities requires concerted efforts at the societal level to promote equitable access to resources, opportunities, and support services for older adults from all walks of life. In conclusion, income plays a multifaceted role in shaping the experiences and outcomes of ageing. While financial resources are not the sole determinant of successful ageing, they serve as a crucial protective factor that enhances overall well-being and resilience in later life. By recognising the importance of income security and addressing systemic barriers to financial access and equity, societies can foster environments where individuals can age with dignity, purpose, and fulfilment [5,18,20,24,25,26,27,29,33,37,40,42,53,54,60].

### 3.20. Engagement in Life

Involvement in various activities (e.g., watching television, looking after grandchildren, engaging in sports) and volunteer work was considered as a measure of engagement with life. The participant’s employment status helped to understand how their engagement with life influenced their perception of ageing well. Retirement significantly impacts how individuals navigate their post-career years and influences their perception of successful ageing. Those who had engaged in retirement planning, including saving and preparing for a healthy older age, had notably different perspectives on their post-career years compared to those who had not made any retirement plans or considered them [5,19,20,21,22,23,24,26,28,29,30,31,32,34,36,41,43,44,48,53,55,56,60,61,65,66].

c. Did previous studies focus more on the theoretical model(s) to study the psychological construct of ageing or did they start from lay perceptions of ageing people?

In the following we will reply to the third research question. In the field of ageing psychology, the evolution of theoretical perspectives and conceptual models has followed a complex path, characterised by a gradual shift from models that followed biomedical and biopsychosocial approaches to the consideration of the lay perspective [5,36,96,109]. 

In the 1950s and 1960s, the biomedical approach dominated the understanding and study of ageing. This approach tended to consider ageing as a process mainly determined by biological and physiological factors, focusing on aspects such as the decline of physical and cognitive functions, chronic diseases, and the incidence of age-related medical conditions. Research mainly focused on identifying risk or prevention factors related to the physical and biological aspects of ageing and treating these signs and symptoms through medical and pharmacological treatments, neglecting psychological, social, and environmental aspects [5,12,26,36,58]. However, in recent decades, this approach has been criticised as increasingly evident evidence suggests that ageing and health are influenced by a wider range of factors and variables. 

One of the main criticisms was its limited consideration of the complexity of the human experience of ageing. By excluding psychological, social, and environmental aspects, the biomedical model did not take into account the influence of emotional factors, social relationships, and psychological adaptation in the experience of successful ageing [26,35,36,40,44,102,110].

Consequently, there emerged the need for a broader and more integrative approach, leading to the conception of the biopsychosocial approach to ageing. The latter developed in the mid-20th century, although its conceptual roots can be traced back to earlier contributions in the history of psychology and medicine [68]. However, it was in the 1970s and 1980s that the biopsychosocial model began to gain more attention and be formalised as an integrated approach to understanding the human experience, including ageing [94,111].

The biopsychosocial model of ageing is a theoretical approach that considers ageing as a process influenced by a complex combination of biological, psychological, and social factors. The biological level considers the physical and physiological aspects of ageing. This includes changes in the functioning of organic systems, the incidence of chronic diseases, the loss of sensory and motor capacities, and other biological processes typical of ageing. The psychological level concerns the mental and emotional aspects of ageing. This includes psychological adaptation to ageing-related challenges, coping strategies, mental health, emotional well-being, and self-perception. Psychological studies explore the cognitive, emotional, and behavioural dimensions of ageing, including factors influencing the quality of life and psychological well-being of the elderly. The social level examines the impact of social relationships, social support networks, family dynamics, cultural norms, and community resources on ageing and the health of the elderly. Social studies explore how social and environmental factors influence the quality of life, social integration, community involvement, and the adaptive capacity of the elderly. This model is based on the idea that ageing and health are the result of dynamic interactions between these different levels of influences [12,94,102,111,112,113,114,115,116]. As described earlier, the Rowe and Kahn model [67,72] can be considered an embodiment of the biopsychosocial model in the field of ageing psychology. Rowe and Kahn introduced the concept of “successful ageing” which goes beyond mere absence of disease and disability to include maintaining good physical and cognitive functioning, as well as active engagement in social life and good quality of life. This model integrates biological (e.g., physical health], psychological (e.g., emotional well-being), and social (e.g., social engagement) elements, recognising the complex interaction among these factors in the overall experience of ageing [67,72].

However, the biopsychosocial approach has also received criticism. Research has highlighted the complexity of effectively integrating the various biological, psychological, and social dimensions of ageing into a single approach. Some authors argue that the biopsychosocial approach tends to overly simplify the complexity and multifinality of ageing, emphasising that it may not fully capture the richness and variety of individual experiences in ageing [22,41,55,117,118]. For example, Fisher [119] emphasised that while the biopsychosocial approach integrates biological, psychological, and social aspects, it risks fragmenting and excessively separating these elements rather than considering them in an integrated way. Furthermore, it highlighted the need for greater attention to the dynamic interaction among different factors in the ageing process. Walker [120] pointed out that the biopsychosocial approach often overlooks the political and economic context in which ageing occurs. It underscored the need to consider social inequalities, access to resources, and the political context as crucial factors influencing the health and well-being of the elderly.

In recent decades, there has been further evolution in how successful ageing is understood and studied, with increasing attention to the lay perspective [22,23,24,26,29,30,31,34,37,38,59].

In psychological research on successful ageing, the lay perspective offers a valuable lens through which to understand people’s perceptions, expectations, and evaluations of ageing. Exploring common perspectives regarding successful, active, healthy, and positive ageing has received less attention than definitions based on theories, as emphasised by Phelan, Anderson, Lacroix, and Larson [55]. However, this type of inquiry is of considerable importance for several reasons. For example, it has significant implications for public policies and public health: when scientific opinions and common ones widely diverge, science-based policies may seem incomprehensible or irrelevant to the public, as observed by Bowling [29]. Conversely, policies and interventions that reflect common conceptions are more likely to receive greater involvement and commitment from the public. Consulting laypeople about their opinions on active ageing can also enrich the scientific discussion and contribute to the development of theoretical models, as emphasised by Montross and colleagues [121] and Strawbridge, Wallhagen, and Cohen [122].

According to the lay perspective, successful ageing is not only about longevity or the absence of disease but rather a complex combination of factors influencing the quality of life in later years. The perception of successful ageing can vary widely from individual to individual and can be influenced by cultural, social, and experiential factors [34,63]. Studies such as Fisher’s [119] have revealed, for example, that social activities are fundamental components of active ageing. Additionally, psychological factors proposed by Ryff [74], such as a sense of purpose and autonomy, have also been mentioned. From a psychological standpoint, perceiving one’s ageing as successful can be analysed through various dimensions. One of these is overall life satisfaction, which includes the perception of personal fulfilment, meaningful relationships, and emotional well-being [29,34,123,124]. Individuals who perceive themselves as having achieved a sense of purpose and meaning in life tend to approach ageing with a more positive and resolute perspective. Furthermore, psychological resilience is a key element in successful ageing. The ability to adapt to challenges and losses associated with ageing, while maintaining a sense of balance and optimism, is crucial for emotional and psychological well-being in old age [12,29,74,92].

The lay perspective on the perception of one’s successful ageing can also be influenced by cultural and social expectations regarding old age. Societies that value experience, wisdom, and the contribution of the elderly tend to have a more positive view of ageing than those that emphasise age as an inevitable decline. Finally, it is important to recognise that successful ageing is a dynamic and subjective concept that can evolve over the course of an individual’s life in response to personal experiences, relationships, and circumstances [107]. Psychological approaches that take into account the lay perspective on successful ageing can offer valuable insights to promote psychological well-being and quality of life in later years. In conclusion, the lay perspective on the perception of one’s successful ageing, explored through a psychological approach, provides us with a richer and more nuanced understanding of what it means to age well. By integrating the experiences, perceptions, and expectations of older adults, we can develop more effective and individual-centred psychological interventions, promoting active, satisfying, and meaningful ageing for all [3,22,23,24,26,29,30,34,37,38,59].

d. Regarding the age of samples, did previous studies focus more on the perception of people in the more advanced phases of life or did they consider the perception of people in all the phases of life to acquire information for both cases? 

With reference to the fourth research question, previous studies on ageing and successful ageing have employed various approaches regarding the age of the samples examined. The Table 3 reports the age groups of the samples used in the fifty articles selected for this scoping review. In particular, the table indicates the minimum age of the subjects selected during the population sampling phases, chosen by the different authors to conduct their studies.

The following table (Table 4) summarises the sampling of participants analysed in the studies selected for this scoping review based on the phases of the life cycle.

Initially, many of these studies primarily focused on the perceptions, experiences, and challenges of individuals in the more advanced stages of life, such as the elderly and seniors aged 65 and above [21,22,23,26,28,29,30,31,33,35,38,50,52,55,56,58,62,63,125]. This approach enabled an understanding of the specific dynamics and unique needs of the elderly population, including considerations of health conditions, social support systems, and economic factors that impact their quality of life.

However, over time, there has been a growing awareness of the importance of considering ageing as a process that involves all stages of life [7,126,127]. This shift in perspective has led to the promotion of research exploring ageing and its implications across the entire lifespan, including individuals of various ages, from younger adults in their 40s and 50s to the elderly [18,19,24,25,27,32,36,39,40,45,46,47,48,49,51,53,54,60,61,64,65,73]. This inclusive approach allows researchers to capture the diverse experiences and transitions that occur across different life stages, from early adulthood through old age.

In contemporary ageing research, it has been recognised that ageing is a complex and multifactorial phenomenon that manifests differently in each stage of life. Therefore, the analysis of ageing processes and factors contributing to successful ageing must take into account the specific experiences and challenges that characterise each phase of human development [9,96]. For example, studies may examine the impact of lifestyle choices, such as diet and exercise, on ageing trajectories across different age groups [128], or explore the role of social relationships and community engagement in promoting resilience and well-being throughout the lifespan [96,129,130].

In this context, the ageing studies which embrace a holistic and longitudinal approach have the ability to provide a more comprehensive view of ageing dynamics and the resources contributing to successful ageing. These approaches allow for the exploration of interconnections between life experiences, biological, psychological, and social factors, as well as environmental changes that influence the ageing process across all stages [96,131].

In conclusion, while initial studies primarily focused on the perceptions of the elderly, contemporary research has expanded its scope to understand ageing as a process involving the entire course of human life, thus offering a richer and more complex perspective on ageing and successful ageing. This broader approach underscores the importance of addressing the diverse needs and experiences of individuals across different age groups to promote healthy ageing and well-being throughout the lifespan [96,107,126,129,131]. 

## 4. Discussion

Ageing is a complex phenomenon which has been studied according to various approaches and perspectives. From a psychological point of view, it represents a phase or more than one phase in the life span. Again, from a psychological point of view, ageing is a phase where learnings, losses, relationships, roles, and challenges are well represented, as in all the other phases of life. Considering the progressive ageing of the population, studying ageing from a psychological point of view means studying how people can age well and with a good quality of life. Even if this topic has been addressed in the last decades, a shared model/definition is not yet available. In this scoping review, and according to the first research question, we addressed all the proposed definitions in the sorted papers, and we found that the concept of successful ageing is the most used and discussed. This concept has undergone significant evolution over time, mirroring shifting societal attitudes, advancements in scientific understanding, and a deeper appreciation for the multifaceted nature of health and well-being [96,129,131]. Examining various models and viewpoints on active and successful ageing reveals a complex interplay of biological, psychological, social, and environmental factors that shape the quality of life for older individuals. The evolution of ageing theories reflects a growing recognition of the diversity and individuality inherent in the ageing process, moving beyond simplistic dichotomies of health versus illness.

Authors such as Havinghurst [68], Rowe and Kahn [67,72], Fernandez-Ballesteros [92], Baltes and Baltes [12,78], Kahana and Kahana [80,101,110], among others, offer diverse perspectives on the biological, psychological, social, and environmental influences on well-being and quality of life. Havinghurst [68] was among the first to introduce the notion of “Successful Ageing”, suggesting that maintaining high levels of activity and life satisfaction in later stages constitutes success. However, early definitions often focused narrowly on biomedical criteria, overlooking the psychological and social dimensions of ageing. Rowe and Kahn [67,72] proposed a model emphasising the absence of disease, physical and cognitive functionality, and engagement in meaningful activities as key components of successful ageing. Despite its merits, this model faced criticism for its narrow focus and lack of attention to socio-cultural contexts and individual challenges in ageing. In response, alternative models like the Positive Ageing Model by Fernandez-Ballesteros and colleagues [92] and Fower, Gaisorek, and Giles’ Communicative Ecology Model of Successful Ageing [70] have emerged, emphasising positive emotional states, social participation, and supportive environments. The World Health Organization (WHO) introduced models stressing health optimisation, participation, and safety for the elderly [71,82]. Productive ageing theories by Butler [93,98,111] emphasised the active contribution of older adults to society, challenging stereotypes of decline and emphasising ongoing growth and engagement. Gergen and Gergen’s Positive Ageing Model [99] highlighted holistic well-being and proactive ageing strategies.

The Selection, Optimization, and Compensation (SOC) Model by Baltes and Baltes [12,78] and the Proactive Coping Model by Kahana and Kahana [80,101,110] offer frameworks for understanding adaptive strategies and resilience in the face of ageing challenges. These models underscore the importance of proactive approaches and leveraging past experiences to enhance well-being.

Overall, these theoretical perspectives underscore the complex interplay of biological, psychological, social, and environmental factors in ageing. Recognising this complexity is crucial for developing interventions and policies that address the diverse needs of ageing populations across different contexts and life stages.

Moving to the second research question, a detailed examination of determinants and predictors in successful ageing reveals critical insights. This is evidenced by the fact that with the gradual ageing of the population, which represents one of the most significant challenges of our time, it becomes necessary and useful to understand the psychological processes that characterise the final phase of the life cycle to design prevention and intervention programs [9,102]. The way these variables and factors interact offers a complex view involving biological, psychological, and social aspects. Socio-demographic variables such as age, gender, education, income, ethnicity, marital status, and place of residence play a fundamental role in ageing studies [5,21,22,23,25,26,32,38,40,41,42,50,55,57,59,61,102].

Determinants, understood as genetic predispositions, cognitive structures, life experiences, and psychosocial influences, form the basis upon which the experience of ageing develops [102]. They shape ageing trajectories and influence individual perceptions of this process [9]. On the other hand, predictors have been defined as crucial tools for anticipating specific outcomes related to ageing, such as mental health, cognitive functioning, and psychosocial adaptation. Among the predictors of successful ageing, education or schooling emerges as a critical factor [23,25,26,28,31,32,34,38,39,40,41,42,43,46,50,52,53,55,58,59,61]. The higher the level of education, the higher the likelihood of maintaining high cognitive functions [104]. Physical activity reducing the risk of cardiovascular diseases and contributing to better mental health [18,19,20,22,23,24,26,27,29,30,32,33,34,38,44,46,47,48,50,51,52,53,55,57,63,79]. Social engagement/involvement offers a sense of belonging, an opportunity to share experiences, and mutual support [5,19,20,21,22,23,24,25,28,30,31,32,34,35,36,38,41,43,44,47,48,53,55,56,60]. 

Moreover, nutrition plays a significant role, with the Mediterranean diet showing associations with a lower incidence of depression and reduced risk of cognitive decline [5,25,26,38,42,47,53,57,60,107].

Spirituality and religiosity can positively influence the well-being of the elderly, offering comfort and a sense of purpose [18,23,25,27,36,38,46,49,53,57,58,59,60,61,64,108].

Autonomy [20,29,33,35,36,40,55] and financial security [5,18,20,24,25,26,27,29,33,37,40,42,53,54,60] are fundamental for satisfactory ageing, allowing the elderly to actively participate in social life and make autonomous decisions regarding their well-being. 

In responding to the third research question, we explore the evolution of perspectives in ageing psychology, observing a notable shift from the dominant biomedical approach to a more inclusive common perspective [5,36,96,109]. Initially, during the 1950s and 1960s, the biomedical approach viewed ageing primarily through the lens of biological and physiological factors, often overlooking psychological and social dimensions [5,12,26,36,58]. However, criticism arose due to its limited grasp of the holistic human experience of ageing [35,40,44,102,110].

The emergence of the biopsychosocial model marked a significant advancement, as it integrated biological, psychological, and social factors into the understanding of ageing [68,94,111]. This model recognised the dynamic interplay among various influences on ageing and health [12,94,111,112,113,114,115,116]. Despite its contributions, critiques surfaced, particularly regarding its tendency to oversimplify the complexity of ageing [22,41,55,117,118].

In recent years, there has been a growing emphasis on the common perspective, which views successful ageing as a multifaceted interplay of various factors beyond the mere absence of disease [22,23,24,26,29,30,31,34,37,38]. This perspective acknowledges the influence of cultural, social, and experiential factors, recognising that successful ageing varies from person to person [34,63].

Furthermore, exploring the lay perspective on successful ageing highlights the disparity between common perceptions and scientific definitions; moreover, it emphasises the need to take into account various perspectives when projecting inclusive policies and interventions in the promotion of satisfying ageing [121,122]. Engaging with the lay perspective can lead to the development of more effective and personalised psychological interventions that to the diverse needs and aspirations of individuals throughout the ageing process.

In addressing our fourth research question, we explored how previous studies on ageing approached the issue of age when selecting samples and determining age cut-offs for studying the ageing process. Initially, many of these studies focused on understanding the perceptions and challenges faced by older individuals, typically aged 65 and above [21,22,23,26,28,29,31,33,35,38,50,52,55,56,58,62,63,125]. However, over time, there has been a growing recognition of the importance of viewing ageing as a lifelong process [7,126,127].

This shift in perspective has prompted researchers to explore ageing across all stages of life, from young adulthood to old age elderly [18,19,24,25,27,32,36,39,40,45,46,47,48,49,51,53,54,60,61,64,65,73]. Nowadays, ageing is acknowledged as a multifaceted phenomenon that unfolds uniquely at each life stage. Consequently, when analysing ageing processes and factors contributing to successful ageing, it is crucial to consider the specific experiences and challenges associated with each phase of human development [9,96].

In essence, while early studies primarily focused on the elderly, contemporary research now embraces ageing as a journey that spans the entire human lifespan. This broader approach offers a more comprehensive understanding of ageing and successful ageing, emphasising the need to address the diverse needs and experiences of individuals across different age groups to promote healthy ageing and overall well-being.

## 5. Conclusions

The conclusion of our investigation into ageing theories, conceptual models according to the perspective of clinical psychology, and research on successful ageing reveals a nuanced understanding of the ageing process. It is no longer simply a matter of physical or biological decline but a complex interplay of various factors influencing the well-being and quality of life of older adults.

We have witnessed a significant evolution in how successful ageing is conceptualised. Initially proposed by Rowe and Kahn in 1987, the concept primarily focused on physical health and cognitive functioning [67]. However, our scoping review illustrates that successful ageing encompasses much more than these aspects.

Today, we recognise successful ageing as a dynamic and multifaceted concept that varies from person to person. It encompasses family relationships, social support, the physical and cultural environment, and a unique blend of elements shaping the quality of life for older adults [96,129].

This broader perspective acknowledges the diversity and individuality of the ageing experience, highlighting that there’s no one-size-fits-all definition of successful ageing. Rather, it comprises multiple facets reflecting life’s complexity.

However, it is crucial to note a significant issue regarding the terminology used in defining successful ageing, particularly in the model proposed by Rowe and Kahn. Our review reveals that the term “success” carries different connotations depending on cultural contexts. In Western contexts, it is often associated with maintaining optimal health, achieving personal and professional goals, and financial stability [3,30,32,33,34,48,49,50,51,55,56,57,58,67,72]. Conversely, in Eastern cultures, successful ageing may emphasise family harmony, respect for elders, wisdom, and community contribution [18,19,20,21,22,23,24,25,26,27].

Considering these cultural nuances is essential to truly capture the essence of successful ageing and represent the entire older population. The Lay Perspective Model emerges as a valuable contribution, suggesting that theorists should incorporate older adults’ perceptions and cultural values into their understanding of successful ageing [22,23,24,25,26,29,31,34,37,38,51,92].

Looking ahead, a promising direction for ageing research involves adopting a mixed-mode approach that combines objective data with subjective experiences. Incorporating the voices and experiences of older adults can inform the development of strategies and interventions to promote satisfying ageing [121,122].

Additionally, there is a growing recognition of ageing as a lifelong process, not limited to old age. Exploring ageing across all life stages allows for a comprehensive understanding of the challenges and opportunities at each phase, enabling the design of more effective interventions [9,96,129].

Moving forward, ageing research should embrace interdisciplinary approaches and continue exploring new horizons to meet the diverse needs of individuals facing ageing and those yet to experience it [96,129].

## Figures and Tables

**Table 1:** Geographical distribution.

Continent	Number of Studies
America	17
Asia	10
Australia	5
Europe	18
Total	50

**Table 2:** Description of sorted papers.

	Authors and Years	Title	Definition/Definitions	Country	Nationality	Semantic/Terminology	Range/Sample Age	Sample Size	Analysed Variables Kind of Variables: Predictors (P) or Determinants (D) if Declared	People Perception 1/External Evaluation 2	Gender %
1	Amin, 2017 [18]	Perceptions of Successful Aging (SA) among Older Adults in Bangladesh: An Exploratory Study	Successful Ageing (Rowe and Kahn, 1987) [67]	Bangladesh, Asia	Bangladeshi	Successful ageing, Aging well	60–90	12	Self-awareness; perception of themselves as old; Financial security; Family and intergenerational care; Social participation	1	58.3% M 41.7% F
2	Balard, 2015 [5]	Old Age: Definitions, Theory and History of the Concept	Ageing Well (Havinghurst, 1961) [68]; Successful Ageing (Rowe and Kahn, 1987) [67]; The disengagement theory (Cumming and Henry, 1961) [69]	France, Europe	ND	Old Age, Ageing well, Successful ageing	ND	ND	Ageing: individual, biological, and social factors	2	ND
3	Bernhold, 2019 [45]	Older Parents’ and Middle-aged Children’s communication as predictors of children’ s successful aging	The communicative ecology model of successful aging (Fowler et al., 2015–2016) [70]; SOC Model (Baltes and Baltes, 1990) [12]	America	Different ethnicities: African America, Asian America, European, America Latina America, Multiethnic, Native America	Aging efficacy, Successful aging	40–50	137	Seven types of language and communication who predict aging efficacy and successful aging: expressing optimism, self-categorising, teasing others about their age, future caregiving preferences, remaining updated, managing being the recipient of ageism, resisting mediated images of ageism	1	38% M 62% F
4	Boruah and Barua, 2021 [19]	Successful Ageing: A study on the senior Teachers of Dibrugarh University, Assam	Successful Ageing (Rowe and Kahn, 1987) [67]; Active Ageing (WHO, 1990) [71]; SOC Model (Baltes and Baltes, 1990) [12]	India, Asia	Indian	Successful Ageing	42–66 Average 54 y	43	Successful Aging Scale	1	81% M 19% F
5	Bosnes et al., 202 [28]	Processing speed and working memory are predicted by components of successful aging: a HUNT study	Successful Ageing (Rowe and Kahn, 1987–1997) [67,72]	Nord-Trondelag Norway, Europe	Norwegian	Successful aging,	70–89	65	Successful ageing: Age, Gender, Education, Absence of disease, High functioning, Engagement with life	1	53.8% F 46.2% M
6	Bowling, 2008 [29]	Enhancing later life: How older people perceive active ageing?	Active Ageing (WHO, 2002) [71]; Successful ageing (Rowe and Kahn, 1987, 1997) [67,72]	Britain, Europe	English	Active Ageing, successful aging	65–74 75+	152 127	Definition of Active Ageing: Having/maintaining physical health and functioning, leisure and social activities, mental functioning and activity, social relationships and contacts psychological, Finances, Independence	1	44% M 56% F
7	Bowling and Iliffe, 2006 [30]	Which model of successful ageing should be used? Baseline findings from a British longitudinal survey of ageing	Successful Ageing (Rowe and Kahn, 1997) [72]	London Britain, Europe	English	Successful ageing	65–70 70–75 75–80 80+	341 281 207 168	Construction of models of successful ageing based on literature: Biomedical model, Broader biomedical model, Social functionating model, Lay models	1	48% F
8	Brown and Bond, 2016 [62]	Comparisons of the utility of researcher-defined and participant-defined successful ageing	Successful Ageing (Rowe and Kahn, 1987) [67]	Adelaide (Australia)	South Australian population	Successful ageing	65–97	380	Successful ageing: Absence of disease, Maintenance of physical and mental functioning, active engagement with life	1	55.8% F
9	Chen et al., 2020 [20]	A self-Reliant Umbrella: Defining Successful ageing among the Old-old (80+) in Shanghai	Successful ageing (Rowe and Kahn, 1987) [67]	Shanghai, Asia	Chinese	Successful Ageing	80–99	97	Successful ageing: Self-reliance, Participating in Physical Activity, Maintaining Financial Security, Social connection, Willingly Accepting Reality	1	56.7% F 43.3% M
10	Chiao and Hsiao, 2017 [21]	Comparison of personality traits and successful aging in older Taiwanese	Successful ageing (Rowe and Kahn, 1987–1997) [67,72]	Taiwan, Asia	Taiwanese	Successful ageing	65–99	174	Successful ageing indicators and personality trait	1	62.1% F 37.9% M
11	Cosco et al., 2015 [31]	Validation of an a priori, index model of successful aging in a population-based cohort study: the successful aging index	Successful ageing (Rowe and Kahn, 1987) [67]	England, Wales, Europe	British, Welsh	Successful ageing	65+	740	Successful Aging Index: Engagement, Personal resources, Cognitive functioning, activities of daily living, Instrumental activities of daily living	1	64.2% F
12	Domenèch-Abella et al., 2018 [32]	Socio-demographic factors associated with changes in successful aging in Spain: a follow-up study	Successful ageing (Rowe and Kahn, 1998) [73]	Spain, Europe	Spanish	Successful ageing	50–99	3625	Indicators of the distinct. SA model: Biomedical, psychosocial, Rowe e Kahn; complete model of SA that included all those indicators	2	53.7% F 46.3% M
13	Gallardo-Peralta and Sanchez-Moreno, 2019 [46]	Successful ageing in older persons belonging to the Aymara native community: exploring the protective role of psychosocial resources	Successful ageing (Rowe and Kahn, 1987) [67]	Chile, America	Aymara ethnicity	Successful Ageing	60+	232	Successful Ageing variable: SAI Successful Ageing Inventory, Community support; Quality of life, Religiousness/spirituality, Mental health, Main health problems	1	65% F 35% M
14	Griffith et al., 2017 [47]	Health is the ability to manage yourself without help: how older African American Men define health and successful aging	Successful ageing (Rowe and Kahn, 1997) [72]	Nashville, America	African Americans	Successful ageing	55–76	22	Rowe e Kahn variables of SA: Autonomy, Functional ability, Imperative to Health, Adherence to Self-care, Definition and social determinants of health	1	100% M
15	Horder et al., 2013 [33]	Self-respect through ability to keep fear of frailty at a distance: Successful ageing from the perspective of community-dwelling older people	Successful Ageing (Rowe and Kahn, 1997) [72]; SOC Model (Baltes and Baltes, 1990 [12]	Western Sweden, Europe	Swedes	Successful ageing	77–90	24	Categories to analysed SA: Having sufficient bodily resources for security and opportunities, Structures that promote security and opportunity, Feeling valuable in relation to outside world, choosing gratitude instead of worries	1	37.5% F 62.5% M
16	Jang et al., 2009 [22]	Association of socioeconomic status with successful ageing: differences in the components of successful ageing	Successful ageing (Rowe and Kahn, 1997) [72]; SOC Model (Baltes and Baltes, 1990) [12]	Seoul, Korea, Asia	Korean	Successful Ageing	65–103	1825	Four components of SA: Physical function, mental function, social function, subjective well-being	2	64.6% F 35.4% M
17	Jopp et al., 2015 [34]	How could Lay Perspective on successful aging complement Scientific theory? Findings from a US and German life-span sample	Successful ageing (Rowe and Kahn, 1997); Ryff (1989) [74]; Baltes and Baltes, (1990) [12]; Depp and Jeste (2006) [75]	Germany Europe, America	American, German	Successful aging, Usual aging, Robust aging, Laypersons’ Perspectives on Successful Aging	15–96	306	Determinants of Successful Aging: Health, Social resources, Activities/interests, Virtues, Attitudes, Beliefs, Well-Being, Life management, Financial resources, Independence, Aging as topic, meaning in life	2	ND
18	Kleineidam et al., 2019 [35]	What is Successful Aging? A psychometric validation study of different construct definitions	Successful Ageing (Rowe and Kahn 1987–1997) [67,72]	Germany, Europe	German	Successful ageing, Subjective successful aging	75+	2478	SA operationalisation: Physiological health, well-being; social engagement SA as a multi-dimensional construct	2	66% F
19	Knight and Ricciardelli, 2003 [63]	Successful aging: perceptions of adults aged between 70 and 101 years	Successful aging (Rowe and Kahn 1987–1997) [67,72]; Disengagement theory (Havighurst, Neugarten e Tobin, 1968) [76]; Theory of gerotrascendence (Maddox, 1968) [77]; Psychological well-being (Ryff, 1989) [74]	Australia	Australian	Successful Aging; Usual aging;	70–101	60	SA components: Health, Happiness, relationships, appreciation/Values of life, Activity, longevity, Independence, Personal growth	2	30% M 70% F
20	Kozerska, 2022 [36]	The concept of Successful Ageing from the perspectives of older adults: an empirical typology	Successful ageing (Rowe and Kahn, 1997) [72]	Poland, Europe	Polish	Successful ageing	60–87	224	Three dimensions of SA: Engagement, Activities, religiousness. Perception of successful ageing, Integrity, spirituality and community	2	61.1% F 38.8% M
21	Lee-Bravatti et al., 2020 [48]	Lifestyle behavioral factors and integrative successful aging among Puerto Ricans living in the mainland United States	Rowe and Kahn (1987, 1997) [67,72]	Massachusetts, America	Puerto Rican	Successful aging	45–75	950	Successful ageing: life satisfaction, social participation, social functioning, psychological resources	2	71.4% F
22	Lewis, 2011 [49]	Successful aging through the eyes of Alaska native elders. What it means to be an elder in Bristol Bay, ak	Successful ageing (Rowe and Kahn, 1987–1997) [67,72]	Bristol Bay Alaska, America	Alaskan	Two different perspectives: successful aging as a state of being, a condition that can be objectively measured a certain moment, successful ageing as a process of continuous adaptation	61–93	26	Four elements of SA: Emotional well-being, Community engagement, spirituality, Psychical Activities	2	61.6% F
23	Li et al., 2006, [23]	Successful aging in Shanghai China: definition, distribution, and related factors	Successful ageing (Rowe and Kahn, 1987) [67]; (Havighurst, 1961) [68]	Shanghai, Asia	Chinese	Successful aging, usual or normal aging	65+	1640	Li et al. criteria of SA: cognitive function, activities of daily living, mood status, and no disability	2	52.9% F
24	Low et al., 2021 [24]	A thematic analysis of older adult’s perspective of successful ageing	Successful ageing (Rowe and Kahn, 1987) [67]; (Baltes and Carstensen, 1996) [78].	Malaysia, Asia	Malay, Chinese, Indian	Successful aging	60–80	12	SA criteria: being healthy, family relationship, financial independent, social connectedness, Being positive	1	58.3% F
25	McCann Mortimer, Winfield, 2008 [64]	Successful ageing by whose definition? Views of older, spiritually affiliated woman	Successful ageing (Rowe and Kahn, 1987, 1997) [67,72]	Adelaide, Australia	Australian	Successful Ageing	60–89	14	Rowe e Kahn approach of SA	2	100% F
26	McLaughlin et al., 2010 [50]	Successful aging in the United States: Prevalence estimates from a national sample of older adults	Successful ageing (Rowe and Kahn, 1987, 1997) [67,72]	United State, America	American	Successful ageing, Healthy aging (Depp and Jeste, 2006) [75]	65+	5177 in 1998 5038 in 2000 5183 in 2002 5414 in 2004	Rowe and Kahn criteria of SA: Active engagement, High cognitive and physical functioning, no major disease	2	59.9% F 40.1% M
27	Molton and Yorkston, 2016 [51]	Growing older with a physical disability: A special application of the successful aging paradigm	Successful ageing (Rowe and Kahn, 1987–1997) [67,72]; Psychosocial approaches of SA (Bowling and Dieppe, 2005) [79]; (Baltes and Baltes, 1990) [12]	United State, America	American	Successful ageing	45–80	49	Criteria SA: Resilience and adaptation, Autonomy, social connectedness, physical health	1	40.9% M 59.1% F
28	Nagalingam, 2007 [25]	Understanding successful aging: a study of older Indian adults in Singapore	Successful ageing (Rowe and Kahn 1998) [72] Stress-theory based model of SA; proactive model (Kahana and Kercher, 1999) [80]	Singapore, Asia	Indian	Successful ageing	60–85	32	Successful ageing determinates in India: Be satisfied in life, high role of activity and educational status Criteria of SA analysed: health status, Life satisfaction, Religion, engagement with life, financial resources and emotional support, Intergenerational transfer and relationship	1	ND
29	Negash et al., 2011 [52]	Successful aging: definitions and prediction of longevity and conversion to mild cognitive impairment	Successful ageing (Rowe and Kahn, 1987) [67]	America	American	Successful and usual ageing, Productive aging, Healthy aging	65+	560	Neuropsychological evaluation of SA into 4 domains: memory, executive faction, language, and visuospatial skills, Age, Associated memory impairment	2	65.7% F
30	Ng et al., 2009 [26]	Determinants of successful aging using a multidimensional definition among Chinese elderly in Singapore	Successful ageing (Rowe and Kahn, 1997) [72]	Hong Kong and Singapore, Asia	Chinese	Successful ageing, Active aging, healthy aging	65+	1281	SA defined by specific sociodemographic, psychosocial, and behavioural determinants	2	60% F 40% M
31	Nguyen and Seal, 2014 [53]	Cross-Cultural Comparison of Successful aging definitions between Chinese of Hmong elders in the United States	Successful ageing (Rowe and Kahn, 1987–1997) [67,72]	Milwaukee, America	Hmong Chinese	Successful aging	60–101 Chinese 61–95	44 (Hmong 21, Chinese 23)	SA domains: Health and wellness, Happiness in old age, financial stability, social engagement, religious faith	2	38.6% M 61.4% F
32	Nosraty et al., 2015 [37]	Perceptions by the oldest old of successful aging, Vitality 90+ Study	(von Faber et al., 2001) [81], Successful aging from two perspective: 1 aging as a state a of being at a certain moment (Rowe and Kahn, 1997) [67,72]; 2 successful aging as a process (Baltes and Baltes, 1990) [12]	Finland, Europe	Finnish	Successful aging, good aging for Finnish	90–91	45	Components of SA: Continuity in the process of aging, Death, harmonious and balanced life, Independence, living circumstances, physical functioning, cognitive functioning and psychological components, social functioning	1	25% F 20% M
33	Pac et al., 2019 [38]	Influence of socialdemographic, behavioral and other health-related factors on healthy ageing based on three operative definitions	Successful aging (Rowe and Kahn, 1997) [72]; Healthy aging (WHO, 2016) [82]	Poland, Europe	Polish	Healthy aging, successful ageing	65+	4653	Healthy aging model based to: functional ability, intrinsic capacity, subjective well-being, health characteristics, genetic inheritance, multimorbidity, need for service, environmental factors	2	51.7% M 48.3% F
34	Paul et al., 2012 [39]	Active Ageing: An empirical approach to the WHO model.	Active Ageing (WHO, 1990s, 2002) [71]; (Bowling, 2008) [29]; Successful ageing (Rowe and Kahn, 1987–1997) [67,72]	Portugal, Europe	Portuguese	Active ageing, Successful ageing, ageing well, healthy ageing, positive ageing	55–101	1322	Active Ageing Model determinants: Personal factors, Behaviour determinants, social environment, health and social services, physical environment, economic determinants Six factors: health components, psychological components, cognitive performance, biological components, social relationship, personality components	2	71.1% F
35	Perales et al., 2014 [40]	Factors associated with active aging in Finland, Poland, and Spain	Active Aging (WHO 2012) [83]; Successful aging (Rowe and Kahn, 1997); Productive aging (Kerschner and Pagues, 1998) [84]; Positive ageing (Bowling, 1993) [85]	Finland, Poland and Spain, Europe	Finnish, Polish and Spanish	Active Aging	50+	10800 Finland 1976; Poland 4071; Spain 4753. Final sample size was 7987	Components of Active Aging: biomedical variables, Psychosocial variables, Social Variables, and external variables	2	57.5% F 42.5% M
36	Peterson et al., 2020 [54]	Healthy ageing in the far north: perspectives	Successful ageing (Rowe and Kahn, 1987, 1997) [67,72]; SOC model (Baltes and Baltes, 1990) [12]	Alaska, America	Alaskans	Successful aging, healthy aging	60–87	30	Components: attitude/perspective, socialisation, sense of community, purpose and staying active, independence, challenges to healthy ageing	1	90% F
37	Phelan et al., 2004 [55]	Older Adults’ Views of Successful aging-How do they compare with researchers’ definitions?	Successful aging (Rowe and Kahn, 1987–1997) [67,72]	America	Japanese American	Successful Aging	65+	1890	Health, physical, functional, psychological and social	1	52.5% F (Jap) 57.7% F (Amer)
38	Plugge, 2021 [41]	Successful ageing in the oldest old: objectively and subjectively measured evidence from a population-based survey in Germany	Successful ageing (Rowe and Kahn, 1997) [72]; SOC model (Baltes and Carstensen, 1996) [78]; two process model (Brandtstadter and Renner, 1990) [86]	North Rhine-Westphalia, Germany, Europe	German	Successful ageing, successful life, Successfulness	80–102	1863	Objective Variants or indicator of SA: absence of disease, physical functioning, cognitive functioning, interpersonal social engagement, productive social engagement Subjective variants of SA: overall life satisfaction, positive and negative aging experience, affective well-being, valuation of life	2 for objective criteria; 1 for subjective criteria	50% M
39	Stewart et al., 2019 [56]	Comparison of self-rated and objective successful ageing in an international cohort	Successful ageing (Rowe and Kahn, 1987) [67]	Canada, America	Canadian, Columbian, Brazilian, Albanian	Successful aging	65–74	1735	SA analysed variables: Physical health, depression, social connectedness, resilience, site	1	53% F
40	Tan et al. 2010 [65]	Experiences of Chinese immigrants and Anglo Australian ageing in Australia	Successful ageing (Havinghurst, 1961) [68]; Successful ageing model (Rowe and Kahn, 1997) [72]	Australia	Anglo and Chinese Australian	Successful ageing	55–78	11 Anglo-Australian; 10 Chinese-Australian	Who do you think it means to age well? What would you make you satisfied in old age? How would ageing in Australia be different to that of your country origin?	1	57.1% F
41	Tan et al., 2011 [66]	Comparing definitions of successful ageing: the case of Anglo- and Chinese Australians	Successful ageing (Rowe and Kahn, 1997) [72]	Australia	Anglo and Chinese Australians	Successful ageing, ageing well, usual ageing,	ND	152 Anglo-Australian; 116 Chinese-Australian	SA questionnaire [55] assesses the views of older people in relation to what they perceive successful ageing, (20 statements). Successful ageing also entailed other psychological and social dimension as adjustments to change, having family and friends	1	ND
42	Tate et al., 2003 [57]	Definition of Successful ageing by elderly Canadian males: The Manitoba follow-up study	Different theories of SA: Rowe and Kahn (1987, 1997) [67,72]; Cuming and Henry (1961) [69]; Ryff (1982) [87]; Hendricks and Hendricks (1986) [88]; Baltes and Baltes (1990) [12]	Manitoba, Canada, America	Canadian	Successful ageing, ageing well, usual ageing	Man age average 78	1821	What is your definition of successful ageing? There are 20 components themes evolved from the respondents’ definitions of successful ageing	1	100% M
43	Tate et al., 2009 [58]	The consistency of definition of successful aging provided by older man: the Manitoba follow-up study	Biomedical or psycho-social models of SA [89]	Manitoba, Canada, America	Canadian	Successful ageing, ageing well	Age average 82 years	First mail 812/1254 questionnaire completed; second mail 870/1216 questionnaire completed	Coding system for definitions of successful ageing has nine main themes: health, health behaviour, having life, productivity, independence, spirituality, acceptance, social networks, life experience	1	100% M
44	Tate et al., 2013 [59]	Older men’s lay definitions of successful aging over time: the Manitoba follow-up study	Two perspectives of definition of SA: clinical models (Rowe and Kahn, 1997) [72]; and psycho-social models (Baltes and Baltes, 1990) [12]	Manitoba, Canada, America	Canadian	Successful ageing, ageing well	Man average 82 years	5898	The SAQ question about physical, mental, and social functioning, living arrangements, retirement and successful ageing What is your definition of successful ageing?	1	100% M
45	Teater and Chonody, 2020 [60]	What attributes of successful ageing are important to older adults? The development of a multidimensional definition of successful aging	Successful aging (Rowe and Kahn, 1987, 1997) [67,72]	America	ND	Successful ageing	55–81	475	SA multidimensional definition includes adapting and coping with life changes, being healthy, having a self-determinants, social relationship, mentally active, personal resources, extrinsic factors Six factors important: social, psychological, physical, financial, environmental, spiritual	1	68.6% F 31.4% M
46	Tyrovolas et al., 2014 [42]	Successful aging, dietary habits and health status of elderly individuals: a k-dimensional approach within the multi-national Medis study	Biomedical models (Rowe and Kahn, 1997) [72]; and psycho-social and Lay models (Bowling and Dieppe, 2006 and Bowling and Iliffe, 2006) [30,79]	Mediterranean island, Europe	European: Italian, Sardinian, Spanish, Greek	Successful ageing	ND	2663	Variables of SA: biomedical, social functioning, subjective models and 10 more components as: education, financial, physical activities, BMI	1	ND
47	Vahia et al., 2011 [61]	Developing a dimensional model for successful cognitive and emotional aging	Successful aging (Rowe and Kahn, 1987) [67]; two factor model of SA, subjective and objective (Pruchno et al., 2010) [90]	San Diego, America	American, Caucasian.	Successful aging	60–89	1948	SA questionnaire; SA variables: Physical/general status, mental/emotional status, cognitive status, psychosocial protective factors, self-rated successful aging	1	100% F
48	Walker, 2002 [43]	A Strategy for active ageing	Successful ageing (Rowe and Kahn, 1987) [67]; Theory of disengagement (Cumming and Henry, 1961) [69]; Productive ageing (Bass, Caro, Chen, 1993) [91]; Healthy ageing (WHO, 2001) [71]	European Country	European	Successful ageing, Productive ageing, Active ageing	ND	ND	Active ageing: promote public health, increase the social support, ensure social protection. Healthy lifestyles, lifelong learning, self-management	ND	ND
49	Whitley et al., 2018 [44]	Population Priorities for successful aging: a randomised vignette experiment	Successful ageing (Rowe and Kahn, 1987/1997) [67,72]	Great Britain, Europe	British	Successful ageing	Six ages groups from 35 to 75	2143	Six dimensions of SA: Disease, disability, physical function, cognitive function, interpersonal engagement, productive engagement	1	ND
50	Zanjari et al., 2016 [27]	Perceptions of Successful ageing among Iranian Elders: insights from a qualitative study	Successful ageing (Rowe and Kahn, 1987/1997) [67,72]; SOC model (Baltes and Baltes, 1990) [12]	Iran, Asia	Iranian	Successful ageing	60+	60	Six determinants of SA: social and psychological well-being, Physical health, Spirituality, financial security, environmental and social context	1	50% F

**Table 3:** Age groups of the samples used in the fifty sorted papers.

Age Minimum Considered	Age Sample AgeMin–AgeMax	Numbers of Papers	Sorted Papers
15	15–96	1	[34]
35	35–75	1	[44]
40	40–66	2	[19,45]
45	45–80	2	[48,51]
50	50+	2	[32,40]
55	55–101	4	[39,47,60,65]
60	60–101	11	[18,24,27,36,46,49,53,54,61,64]
65	65–103	13	[21,22,23,26,29,30,31,38,50,52,55,56,62]
70	70–101	3	[28,58,63]
75	75–not specified	1	[35]
77	77–90	1	[33]
80	80–not specified	4	[20,41,58,59]
90	90–91	1	[37]
ND	Not analysed	4	[5,42,43,66]

**Table 4:** Sampling of participants in the sorted papers.

Stage of Life	Range of Years	Age Sample Age Min–Age Max	Number of Papers	Sorted Papers
Adolescence to adulthood	15–40	15–96	2	[34,44]
Advanced Adulthood	40–64	40–101	21	[18,19,24,25,27,32,36,39,40,45,46,47,48,49,51,53,54,60,61,64,65]
Old Age/Elderhood	65–79	65–103	18	[21,22,23,26,28,29,30,31,33,35,38,50,52,55,56,58,62,63]
Oldest Old/Advanced Elderhood	80+	80–91	5	[20,37,41,58,59]
	ND	Not analysed	4	[5,42,43,66]
